# Effect of bisphosphonate treatment in osteogenesis imperfecta children in Cipto Mangunkusumo Hospital Jakarta

**DOI:** 10.1186/1687-9856-2015-S1-P61

**Published:** 2015-04-28

**Authors:** Aman Pulungan, Jose Batubara, Bambang Tridjaja, Frida Soesanti, Margaret Zacharin, Putri Alevia, Dwi Lestari Pramesti

**Affiliations:** 1 University of Indonesia, Jakarta, Indonesia; 2 Cipto Mangunkusumo Hospital, Jakarta, Indonesia; 3 Indonesia Pediatric Society, Jakarta, Indonesia; 4 Royal Children’s Hospital, Melbourne, Australia

**Keywords:** osteogenesis imperfecta, biphosphonates, treatment

## Background

Bisphosphonates are the mainstay of pharmacologic fracture-prevention therapy for most forms of osteogenesis imperfecta (OI). Studies suggests that bisphosphonate treatment may significantly improve the natural history of all type of OI, particularly by decreasing the rate of fracture, increasing bone mineral density, decreasing bone pain, and significantly increasing height.

## Objective

To evaluate the effect of bisphosphonate treatment in OI children treated in Cipto Mangunkusumo Hospital (CMH) Jakarta.

## Method

We retrospectively studied the data of age, gender, age at diagnosis, bisphosphonate treatment, and its effects in OI patients in CMH from Indonesian OI children registry, which is documented from January 2012 to May 2014.

## Results

Seventy-seven OI cases (39 male), which were diagnosed at the age of 1 - 9 years old were recorded. Five patients underwent surgery and three patients died before commencing treatment. Nine patients received intravenous pamidronate and sixteen patients received zolendronic acid therapy. Liver and renal functions, as well as serum electrolyte levels were evaluated before and after treatment. Eight patients reported hyperthermia and three others experienced fatigue, bone pain, and abdominal pain within 24 hours of therapy. Serum calcium level decreased in eight patients. No serious adverse effects were documented.

## Conclusions

All registered OI cases patients that received bisphosphonates treatment showed no serious adverse effects.

